# The swim-and-sink behaviour of copepods: a revisit to mechanical power requirement and a new hypothesis on function

**DOI:** 10.1098/rsos.230347

**Published:** 2023-07-12

**Authors:** Houshuo Jiang

**Affiliations:** Applied Ocean Physics and Engineering Department, Woods Hole Oceanographic Institution, Woods Hole, MA 02543, USA

**Keywords:** copepod fluid dynamics, swim-and-sink behaviour, detection and capture of diatom chains, *Centropages* sp., *Temora* sp.

## Abstract

Many copepods display a swim-and-sink behaviour, which is not energetically efficient but probably aids in perceiving and capturing diatom chains. Here, computational fluid dynamics was employed to calculate the mechanical power required by a negatively buoyant, self-propelled copepod in swim-and-sink versus hovering. The results show that upward swim-and-sink about a fixed depth always demands more power than hovering. Subsequently, high-speed microscale imaging was employed to observe the copepod *Centropages* sp. in swim-and-sink, specifically its encounter and handling of diatom chains for capture, along with the measured alternating swimming and sinking currents imposed by the swim-and-sink copepod. The findings suggest that during upward swimming, the copepod uses its swimming current to scan the fluid for detecting embedded diatom chains, presumably through chemoreception. Once a diatom chain is perceived, the copepod sinks and uses its sinking current to manipulate the orientation of the diatom chain before swimming upward to capture it. Overall, these results propose a hypothesis that swim-and-sink is an innate behaviour that assists copepods in perceiving and manoeuvring diatom chains for capture. In contrast with near-spherical algae, diatom chains predominately exhibit a horizontal orientation in the ocean, necessitating vertically oriented copepods to possess a handling behaviour that manoeuvres diatom chains for capture.

## 1. Introduction

Marine planktonic copepods usually dominate the abundance and biomass of marine zooplankton [1–3]. They play essential roles in marine food webs as important consumers of phytoplankton as well as other protist and animal prey, and as important prey for higher level consumers such as fish larvae and fish, arrow worms, krill and suspension-feeding whales (e.g. [2,4,5]). They also contribute significantly to ocean carbon cycling through such mechanisms as producing fecal pellets, moults and carcasses that sink through the water column and performing diel vertical migrations [6,7]. Because of the importance of copepods, numerous studies have been conducted to shed light on the biology, physiology, ecology, oceanography, biological–physical interactions and ecological fluid dynamics of copepods.

Copepods display a variety of free-swimming behaviours, including hovering, upward swimming, free sinking, partial sinking and horizontal backward or forward swimming (see [2], table 1 of [8] and [9] for reviews). Copepods use these behaviours to generate feeding, swimming and sinking currents that are important for their feeding, sensing and signalling [10–17]. Copepods also modulate a suitable sequence of their swimming behaviours, thereby generating a spatio-temporally varying flow; this unsteady feeding current is energetically more efficient than a constant feeding current of a wider range, because it functions to entrain those water parcels containing algae and leave behind those without valuable food in them [9].

Being negatively buoyant, quite a few species of copepods, e.g. *Calanus finmarchicus*, *Centropages typicus*, *Ce. velificatus* and *Paracalanus parvus*, perform sequences of upward swimming for a short distance followed by passive sinking [12,14,18–22], called the ‘swim-and-sink’ behaviour. So far, the function of the swim-and-sink behaviour is unclear.

A theoretical study examined the mechanical power requirement of the upward swim-and-sink behaviour in comparison with hovering [23]. The study concluded that under certain conditions a negatively buoyant copepod that performs an upward swim-and-sink manoeuvre requires less power than that the same but otherwise hovering copepod requires. The study, however, was based on two flawed assumptions: (i) the drag force acting on the copepod was expressed by a drag law using squared velocity, and (ii) a ‘virtual hovering velocity’ was introduced such that the excess weight was expressed also by a drag law using squared ‘virtual hovering velocity’. The first assumption allowed calculating the drag force using a drag coefficient; however, the study used the drag coefficient for a towed body, which is not suitable for a self-propelled swimming copepod. In the second assumption, the excess weight of a negatively buoyant copepod was interpreted to be equivalent to an otherwise neutrally buoyant copepod swimming upward into a current speed of the ‘virtual hovering velocity'; when the neutrally buoyant copepod was swimming at the ‘virtual hovering velocity', it became hovering in an inertial frame of reference. This interpretation was problematic because hovering should not be simply treated by a reference transformation [16].

Therefore, this study revisited the mechanical power requirement of the upward swim-and-sink behaviour versus hovering. To be specific, a computational fluid dynamics (CFD) simulation approach was used to compute the flow field generated by a negatively buoyant, self-propelled copepod swimming steadily upward at a constant speed. The flow field around a hovering copepod (i.e. at zero swimming speed) was also simulated. These flow field simulations were the building blocks for an ensuing CFD-based energetic analysis of the swim-and-sink behaviour. The mechanical power consumption was calculated for each simulated swimming behaviour, based on the CFD-simulated flow field. Then, the mechanical power consumption of the upward swim-and-sink behaviour was mapped for several realistic combinations of copepod body size and excess weight and compared with hovering. It was found that upward swim-and-sink about a fixed depth always requires more mechanical power than hovering does. Now that the swim-and-sink behaviour is energetically costly, what has it been adapted for? To shed light on this question, a high-speed microscale imaging system (HSMIS) was used to observe the upward swim-and-sink behaviour of the copepod *Centropages* sp. in detail, particularly, the encounter and capture of diatom chains by the copepod. Based on these observations, a new hypothesis was proposed that the upward swim-and-sink behaviour may aid the copepod to detect and capture diatom chains more effectively, considering that diatom chains show preferential horizontal orientation in the ocean [24].

## 2. Material and methods

### 2.1. Computational fluid dynamics-based energetic analysis

**Figure 1.**
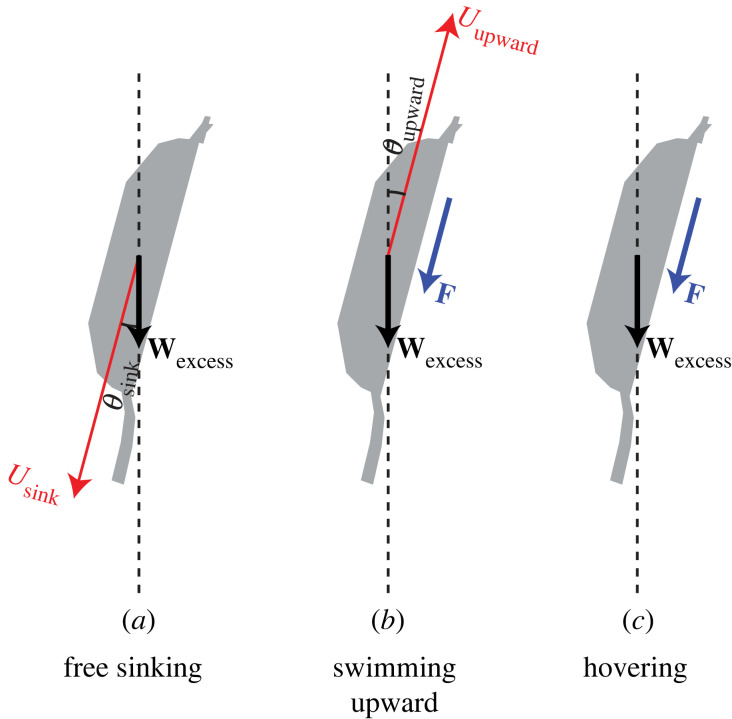
Three copepod swimming modes simulated by CFD. The model copepod sinks freely at speed *U*_sink_ and direction angle *θ*_sink_ (*a*), swims upward at speed *U*_upward_ and direction angle *θ*_upward_ (*b*) or hovers (*c*). Denote **D** (a vector) the hydrodynamic drag acting on the copepod body (not drawn), **T** (a vector) the thrust acting on the copepod body (not drawn), which is the reaction force to the propulsive force **F** (a vector) that the cephalic appendages exert on the water, and **W**_excess_ (a vector) the excess weight, then **D** + **W**_excess_ = **0** holds in (*a*), and **T** + **D** + **W**_excess_ = **0** holds in (*b*) and (*c*).

The analysis of mechanical power consumption of copepod swim-and-sink behaviour was based on CFD simulation results. To be specific, a CFD simulation approach has been previously developed to compute the flow field imposed by a negatively buoyant, self-propelled copepod that swims at a constant speed and direction and the required mechanical power [9,25]; the approach is recapitulated in the electronic supplementary material, section S1. In this study, a model copepod of a given body shape, prosome length *L*, and excess weight *W*_excess_ was considered, and the CFD approach was used to determine: (i) the sinking speed *U*_sink_ and direction angle *θ*_sink_ of the model copepod when it sinks freely (figure 1*a*), (ii) the direction angle *θ*_upward_ of the model copepod when it swims upward at speed *U*_upward_ (figure 1*b*) and the required mechanical power *P*_upward_, and (iii) the mechanical power *P*_hover_ required by the model copepod when it hovers (figure 1*c*). Then, the model copepod was assumed to maintain a fixed mean depth by a repeated upward swimming at speed *U*_upward_ and direction angle *θ*_upward_ for a period *t*_upward_ followed by free sinking at speed *U*_sink_ and direction angle *θ*_sink_ for a period *t*_sink_. Thus, the required mechanical power *P*_swim−and−sink_ was calculated as follows:

2.1 $$P_{\mathrm{swim}-\mathrm{and}-\mathrm{sink}}=\frac{P_{\mathrm{upward}}}{1+(U_{\mathrm{upward}}\cos(\theta_{\mathrm{upward}})/U_{\mathrm{sink}}\cos(\theta_{\mathrm{sink}}))},$$

and then compared with *P*_hover_. Four sets of simulation examples were performed with the parameters—the prosome length *L* and the excess weight *W*_excess_ (i.e. the excess density Δ*ρ*)—being within the typical ranges for calanoid copepods (table 1). Most copepod species have an *L* of 0.5–5.0 mm (fig. 3 of [2]), and Δ*ρ* of copepods should fall in the range of 0–100 kg m^−3^ [2,26].

**Table 1.** Summary of four sets of CFD simulation examples.

model copepod parameters	upward swimming speed *U*_upward_ (mm s^−1^)	upward swimming direction angle *θ*_upward_ (°)	required mechanical power *P*_upward_ (W)
prosome length *L* = 0.67 mm; excess weight *W*_excess_ = 5.169 × 10^−9^ N (excess density Δ*ρ* = 23.1 kg m^−3^); free sinking speed *U*_sink_ = 0.6 mm s^−1^; free sinking direction angle *θ*_sink_ = 0.381°; required mechanical power for hovering *P*_hover_ = 1.199 × 10^−10^ W	0.15	0.675	1.945 × 10^−10^
	0.3	1.110	2.871 × 10^−10^
	0.45	1.344	3.972 × 10^−10^
	0.6	2.029	5.249 × 10^−10^
	0.75	2.629	6.700 × 10^−10^
	0.9	3.060	8.322 × 10^−10^
	1.05	3.908	1.011 × 10^−9^
	1.2	4.856	1.205 × 10^−9^
prosome length *L* = 1.135 mm; excess weight *W*_excess_ = 7.118 × 10^−9^ N (excess density Δ*ρ* = 6.5 kg m^−3^); free sinking speed *U*_sink_ = 0.509 mm s^−1^; free sinking direction angle *θ*_sink_ = 0.389°; required mechanical power for hovering *P*_hover_ = 1.789 × 10^−10^ W	0.12725	1.194	2.796 × 10^−10^
	0.2545	1.865	4.020 × 10^−10^
	0.38175	2.571	5.424 × 10^−10^
	0.509	3.479	7.042 × 10^−10^
	0.63625	4.474	8.827 × 10^−10^
	0.76345	5.582	1.079 × 10^−9^
	0.89075	6.778	1.292 × 10^−9^
	1.018	8.056	1.521 × 10^−9^
prosome length *L* = 1.2 mm; excess weight *W*_excess_ = 9.716 × 10^−8^ N (excess density Δ*ρ* = 75.5 kg m^−3^); free sinking speed *U*_sink_ = 5.5 mm s^−1^; free sinking direction angle *θ*_sink_ = 0.898°; required mechanical power for hovering *P*_hover_ = 1.907 × 10^−8^ W	2.75	8.240	2.903 × 10^−8^
	5.5	9.138	4.340 × 10^−8^
	8.25	9.747	6.200 × 10^−8^
	11	10.143	8.457 × 10^−8^
		
		
		
		
prosome length *L* = 2.5 mm; excess weight *W*_excess_ = 2.366 × 10^−8^ N (excess density Δ*ρ* = 2.0 kg m^−3^); free sinking speed *U*_sink_ = 0.75 mm s^−1^; free sinking direction angle *θ*_sink_ = 0.546°; required mechanical power for hovering *P*_hover_ = 8.539 × 10^−10^ W	0.375	5.333	1.721 × 10^−9^
	0.75	8.666	2.741 × 10^−9^
	1.125	11.688	3.910 × 10^−9^
	1.5	14.028	5.243 × 10^−9^
		
		
		
		

### 2.2. Computational fluid dynamics-based drag analysis

In the present CFD simulations, the hydrodynamic force (a vector) acting on the model copepod was calculated as the area integral of pressure and shear stress over the body surface of the model copepod. The body drag *D* was obtained by projecting the calculated hydrodynamic force along the swimming, sinking or towing direction. These calculations were done for cases of both a towed copepod body and a self-propelled copepod. Then, for the cases of a towed copepod body, the drag coefficient *C*_D_ was calculated as follows:

2.2 $$C_{D}=\frac{\left|D\right|}{(1/2)\rho A{\left|U\right|}^{2}},$$

where *ρ* is the density of the fluid, *U* the towing velocity and the cross-sectional area $A=\pi r_{e}^{2}$ with the volume-equivalent-sphere radius $r_{e}={\left(3V_{\mathrm{copepod}}/4\pi \right)}^{1/3}$ where *V*_copepod_ is the body volume of the model copepod. Also, the Reynolds number Re was calculated for all cases as follows:

2.3 Re=|U|2reμ/ρ,

where *µ* is the dynamic viscosity of the fluid and *U* the swimming, sinking, or towing velocity.

For a self-propelled copepod, a scaling relationship was obtained relating the body drag *D* to both the excess weight *W*_excess_ of the model copepod and the drag coefficient *C*_D_ of the model copepod when it is towed. Specifically, the scaling relationship was determined by fitting data obtained from CFD simulations that varied systematically three key parameters, namely, the size of the model copepod, the excess density of the model copepod (Δ*ρ* = *ρ*_copepod_−*ρ*, where *ρ*_copepod_ is the mass density of the model copepod), and the upward swimming, partial sinking or free sinking velocity *U* of the model copepod.

### 2.3. Experiments

Copepodites and adults of the copepod *Centropages* sp. were collected using a plankton net (50 cm mouth diameter, 3 : 1 length-to-mouth ratio and 335 µm mesh size) in February and March 2016 from a pier in Woods Hole, Massachusetts, USA. The seawater temperature was approximately 4°C. In the laboratory, copepods were sorted and, together with naturally co-occurring algae, acclimatized at room temperature (either approx. 17 or 20°C). Maintained at either acclimatization temperature, the copepods were observed within 48 h of their collection.

The HSMIS was used to observe the upward swim-and-sink behaviour of *Centropages* sp., including the encounter and capture manoeuvre by the copepod toward diatom chains. The HSMIS approach has been described adequately in previous studies that used it to observe protists [27–30], copepod adults, copepodites and nauplii [31,32], and small benthic suspension feeders [33,34]. The optical set-up specific to this study consisted of an objective lens of 150 mm focal length and an infinity-corrected, long-working-distance microscope objective (4 × /0.10 18.5 mm working distance). This lens combination was mounted horizontally to a Photron FASTCAM SA3 120 K monochrome video camera (1024 × 1024-pixel image resolution at 2000 frames per second (fps)), resulting in a vertically oriented field-of-view of approximately 4.8 × 4.8 mm. A glass cell (Hellma Large-Cuvette 740.000-OG, 100 ml, 34.5 mm light path) held approximately 20 copepodite and adult copepods, together with naturally co-occurring algae as food. (The source, species composition and density of algae were not determined.) The glass cell was placed properly such that the microscope objective was focused on the centre of the glass cell, thereby minimizing the wall effect. A collimated 1 W white LED light source provided backlit illumination; only the field-of-view was illuminated by using an iris diaphragm to block the unwanted outskirt light; the resulted weak lighting appeared not affecting the behaviour of the copepod. In total, seven rounds of observations were conducted on multiple days.

Time-resolved micro-particle image velocimetry (TR-μPIV) was used to measure the time-dependent flow field imposed by *Centropages* sp. in the upward swim-and-sink behaviour. This was done by adding 3 µm diameter polystyrene particles as tracer particles to the glass cell and using the HSMIS to take high-speed brightfield videos. Despite using volume illumination, the HSMIS was equipped with a high-magnification optical set-up of a narrow depth-of-focus (DoF approx. 151 µm for the present set-up; electronic supplementary material, section S2). Thus, a well-focused thin slice of tracer particles in the copepod flow field was imaged, thereby enabling two-dimensional µPIV (e.g. [35]); the thin slice cut through the copepod body along either a ventral/dorsal plane or a lateral plane. The original videos were taken at 2000 fps. It turned out that the time interval between two consecutive images was too short to allow sufficient displacements of tracer particles. To remedy this problem, the original videos were subsampled at 200 fps for the µPIV analysis. Next, all subsampled brightfield videos were inverted via the ImageJ software and then imported into the DaVis v. 8.40 software (LaVision) for PIV processing. Additionally, an intensity threshold-based mask was applied to the pixels of the copepod body, thereby excluding them from PIV vector calculations. Cross-correlation of two consecutive images, the so-called ‘single frame’ mode, was used to calculate the velocity vector field. Specifically, a multi-pass iteration algorithm was applied using initial and final interrogation window sizes of 32 × 32 and 16 × 16 pixels (75 × 75 µm), respectively, with a 50% overlap.

All videos (including the ones with tracer particles) were imported into the ImageJ software for measuring copepod prosome length *L*, free sinking time *t*_sink_ and speed *U*_sink_, upward swimming time *t*_upward_ and speed *U*_upward_, and appendage beat frequency *f* during upward swimming. Videos were also inspected for describing the encounter and capture manoeuvre by the copepod toward diatom chains. Note that the original 2000 fps videos were used for these analyses.

## 3. Results

### 3.1. Computational fluid dynamics-derived mechanical power

**Figure 2.**
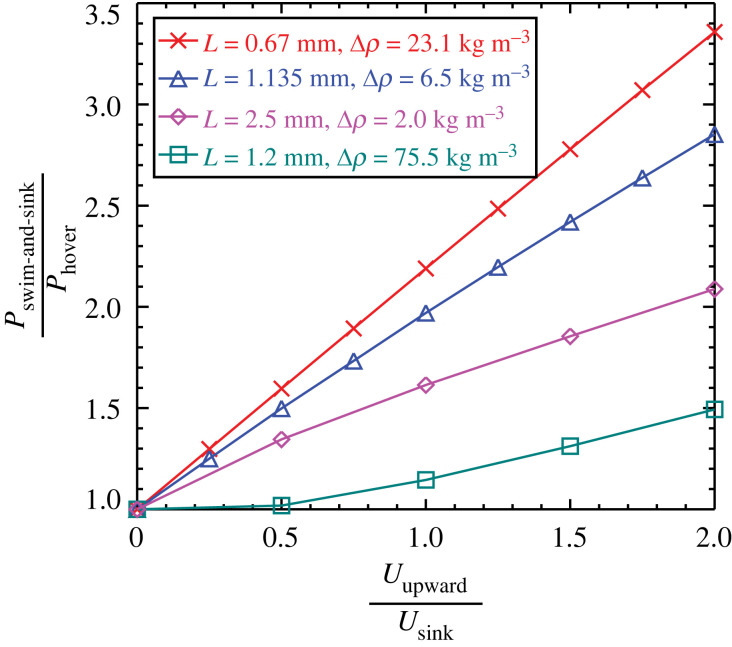
*P*_swim−and−sink_ / *P*_hover_ plotted against *U*_upward_ / *U*_sink_ for four sets of CFD simulation examples.

Based on data derived from CFD simulation examples (table 1), the power ratio *P*_swim−and−sink_ / *P*_hover_ was plotted against the speed ratio *U*_upward_ / *U*_sink_ (figure 2). *P*_swim−and−sink_ / *P*_hover_ is always greater than 1 and increases monotonically with increasing *U*_upward_ / *U*_sink_. Thus, the CFD-derived mechanical power requirement of the swim-and-sink behaviour is greater than that of hovering.

### 3.2. Comparison of drag between a towed copepod body and a self-propelled copepod

**Figure 3.**
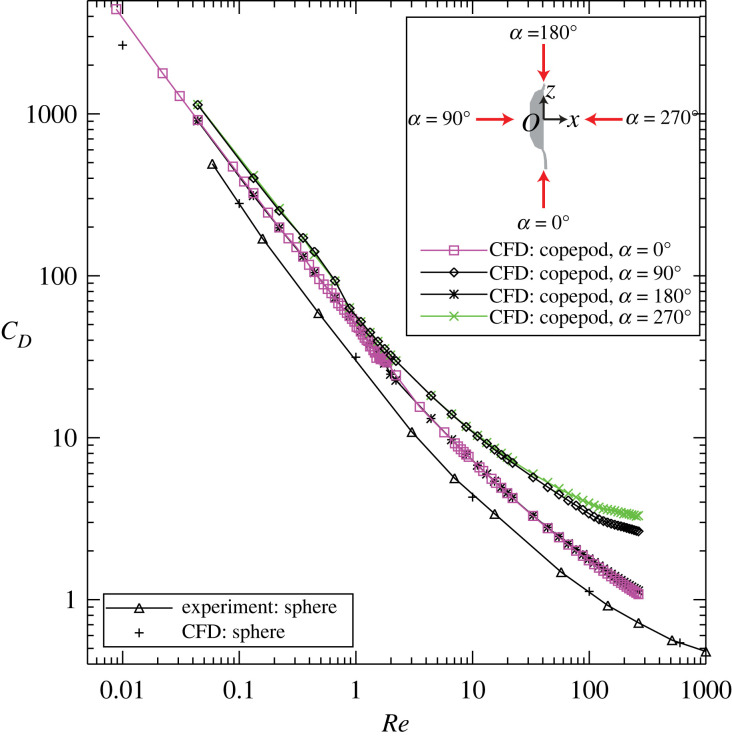
The drag coefficient *C*_D_ plotted against the Reynolds number Re, for a towed copepod (for which four towing directions were considered in CFD simulations) and for a towed sphere (for which both experiment- and CFD-derived data were plotted).

In the present CFD simulations, four towing directions were considered for a towed copepod, and *C*_D_s were calculated and compared with each other and with that of a towed sphere (figure 3). A posteriorly towed copepod (figure 3 inset, *α* = 0°) and an anteriorly towed copepod (*α* = 180°) have similar *C*_D_s, while a dorsally towed copepod (*α* = 90°) and a ventrally towed copepod (*α* = 270°) have similar *C*_D_s. At the same Re, the posteriorly or anteriorly towed copepod has a smaller *C*_D_ than the dorsally or ventrally towed copepod does. Also, all four *C*_D_s obtained for a towed copepod are larger than that of a towed sphere. It is noted that these CFD-derived *C*_D_s for a towed copepod are similar to those used by Haury & Weihs [23] (their fig. 1) for calculating the mechanical energy expenditure by a swim-and-sink copepod, which was in fact modelled as a towed body in their study.

**Figure 4.**
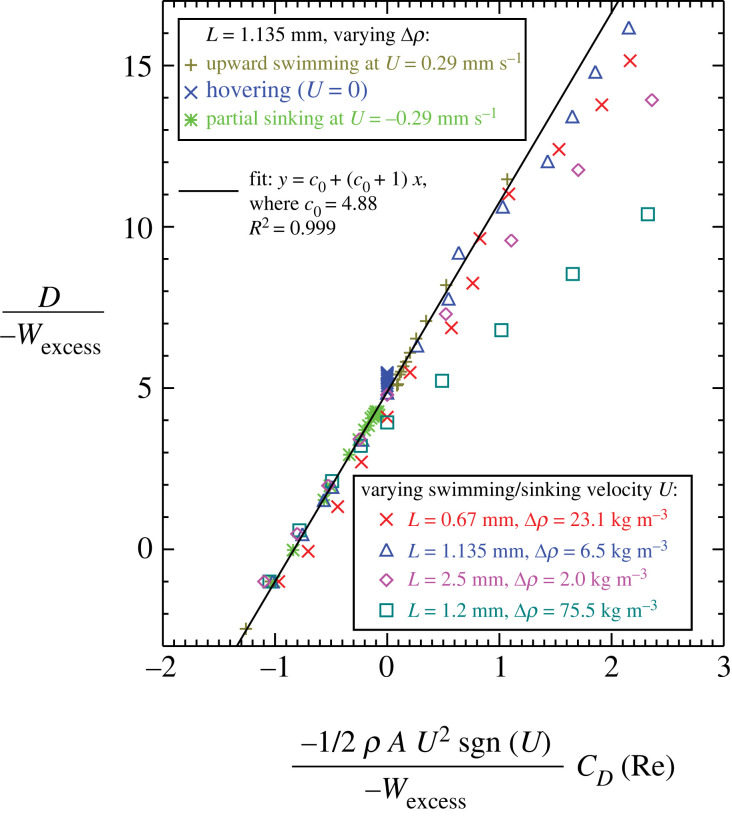
Scaling relationship relating the body drag *D* to both the excess weight *W*_excess_ of the model copepod and the drag coefficient *C*_D_(Re) of the model copepod when it is towed, where *C*_D_(Re) denotes that *C*_D_ is a function of Re and sgn(*U*) is the sign function of *U*. The fitting data were derived from CFD simulations of systematically varying key parameters (see figure insets for detail).

For a self-propelled copepod in steady upward swimming/hovering/partial sinking/free sinking, the CFD-derived scaling relationship can be written as follows:

3.1 D=−4.88Wexcess−5.88ρAU2sgn(U)2CD(Re),

which relates the body drag *D* to both the excess weight *W*_excess_ of the model copepod and the drag coefficient *C*_D_(Re) of the model copepod when it is towed (figure 4). By contrast, the drag force acting on a towed copepod can be written as follows:

3.2 Dtowed=ρAU22CD(Re).

In equation (3.1), *W*_excess_ = Δ*ρ g V*_copepod_ with *g* the gravitational acceleration; when *W*_excess_ > 0 the copepod is negatively buoyant (i.e. heavier than seawater) and when *W*_excess_ < 0 the copepod is positively buoyant (i.e. lighter than seawater). *C*_D_(Re) denotes that *C*_D_ is a function of Re for a towed copepod body, (i.e. equations (2.2) and (2.3) and figure 3); given a value of Re, *C*_D_ is calculated via interpolation using the curve of either a posteriorly or anteriorly towed copepod (figure 3) depending on the moving direction of the self-propelled model copepod. sgn(*U*) is the sign function of *U*, where sgn(*U*) = −1 for *U* < 0 (i.e. partial sinking or free sinking), sgn(*U*) = 0 for *U* = 0 (i.e. hovering), and sgn(*U*) = 1 for *U* > 0 (i.e. upward swimming). It is noted that equation (3.1) reduces to *D* = *W*_excess_ for a free sinking copepod.

The stark difference between equation (3.1) and equation (3.2) suggests that it is quantitatively incorrect to use the drag equation for a towed copepod, i.e. equation (3.2), for calculations concerning a self-propelled copepod. This conclusion is not surprising because a towed copepod and a self-propelled copepod differ significantly in their imposed flow velocity vector and pressure fields around their body (electronic supplementary material, figure S2).

### 3.3. Swim-and-sink behaviour and kinematics of *Centropages* sp.

Individuals of *Centropages* sp. were observed to display the swim-and-sink behaviour that typically consists of a sequence of upward swimming for a short distance followed by passive sinking (electronic supplementary material, video group S1). In natural seawater at 20°C, individuals of *Centropages* sp., 0.95 ± 0.15 mm (*n* = 24) in prosome length *L*, beat their cephalic appendages at 52 ± 9 Hz (*n* = 35) to swim upward at 5.07 ± 3.79 mm s^−1^ (5.06 ± 3.38 *L* s^−1^; *n* = 35) for 0.2413 ± 0.1537 s (*n* = 26), interrupted by sinking freely at 2.05 ± 0.54 mm s^−1^ (2.08 ± 0.43 *L* s^−1^; *n* = 48) for 0.5310 ± 0.3352 s (*n* = 27) (table 2*b*).

**Table 2.** Summary of swim-and-sink kinematics of the copepod *Centropages* sp.

	*L* (mm)	*t*_sink_ (s)	*U*_sink_ (mm s^−1^)	*U*_sink_ (*L* s^−1^)	*t*_upward_ (s)	*U*_upward_ (mm s^−1^)	*U*_upward_ (*L* s^−1^)	*f* (Hz)
(*a*) in natural seawater at 17°C:								
mean ± s.d.	1.05 ± 0.13	0.3848 ± 0.2271	2.08 ± 0.49	1.99 ± 0.40	0.2565 ± 0.1480	4.67 ± 1.81	4.45 ± 1.59	47 ± 7
range	0.85–1.24	0.1375–0.8425	1.03–3.65	1.12–3.41	0.0975–0.6815	1.04–8.26	1.22–7.44	38–67
*n*	15	15	30	30	20	27	27	27
(*b*) in natural seawater at 20°C:								
mean ± s.d.	0.95 ± 0.15	0.5310 ± 0.3352	2.05 ± 0.54	2.08 ± 0.43	0.2413 ± 0.1537	5.07 ± 3.79	5.06 ± 3.38	52 ± 9
range	0.67–1.15	0.1155–1.2745	0.99–3.02	1.09–3.25	0.0670–0.7335	0.70–20.60	0.76–17.91	36–69
*n*	24	27	48	48	26	35	35	35
(*c*) in natural seawater seeded with 3 µm diameter polystyrene particles at 20°C:								
mean ± s.d.	0.95 ± 0.15	0.5610 ± 0.2976	1.73 ± 0.42	1.84 ± 0.33	0.3276 ± 0.3274	6.42 ± 2.10	6.72 ± 1.45	57 ± 7
range	0.67–1.17	0.2165–1.1440	1.31–2.46	1.23–2.38	0.1170–1.2840	3.27–9.92	4.39–9.31	45–69
*n*	17	13	28	28	11	24	24	25

**Figure 5.**
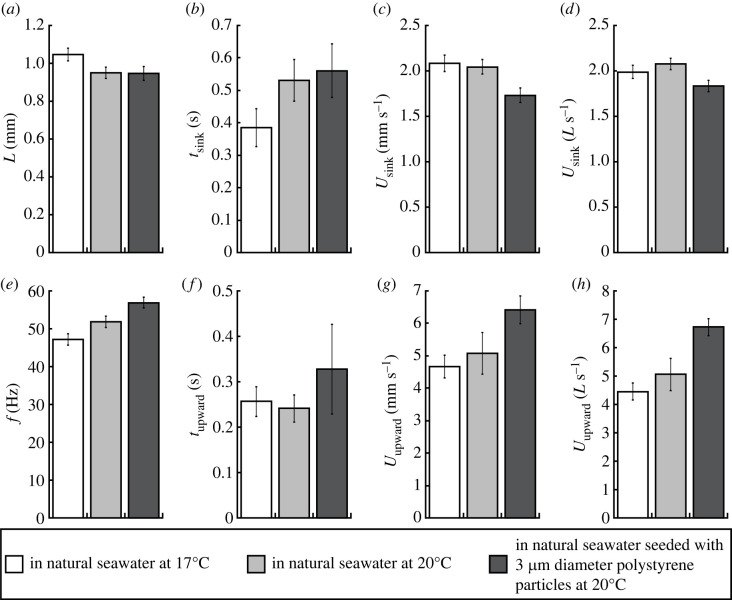
Copepod prosome length *L* (*a*), free sinking time *t*_sink_ (*b*), free sinking speed *U*_sink_ in both mm s^−1^ (*c*) and *L* s^−1^ (*d*), appendage beat frequency *f* during upward swimming (*e*), upward swimming time *t*_upward_ (*f*) and upward swimming speed *U*_upward_ in both mm s^−1^ (*g*) and *L* s^−1^ (*h*) for three observation conditions: (i) in natural seawater at 17°C, (ii) in natural seawater at 20°C and (iii) in natural seawater seeded with 3 µm diameter polystyrene particles at 20°C. Error bars represent s.e.

Observations were also made for *Centropages* sp. in natural seawater at 17°C (table 2*a*) and in natural seawater seeded with 3 µm diameter polystyrene particles at 20°C (table 2*c*). Thus, the effects due to the two different temperatures or the presence of small particles were analysed (figure 5). With increasing temperature from 17°C to 20°C, *t*_sink_ increased (0.3848 ± 0.2271 s versus 0.5310 ± 0.3352 s) but not significantly (Student's *t*-test, *p* = 0.1016), *U*_sink_ did not change significantly (1.99 ± 0.40 *L* s^−1^ versus 2.08 ± 0.43 *L* s^−1^; *p* = 0.3472), *f* increased significantly (47 ± 7 Hz versus 52 ± 9 Hz; *p* = 0.03054), *t*_upward_ did not change significantly (0.2565 ± 0.1480 s versus 0.2413 ± 0.1537 s; *p* = 0.7364), and *U*_upward_ increased slightly (4.45 ± 1.59 *L* s^−1^ versus 5.06 ± 3.38 *L* s^−1^) but not significantly (*p* = 0.3543). At 20°C in the absence versus in the presence of 3 µm diameter polystyrene particles, *t*_sink_ did not change significantly (0.5310 ± 0.3352 s versus 0.5610 ± 0.2976 s; *p* = 0.7771), *U*_sink_ decreased significantly (2.08 ± 0.43 *L* s^−1^ versus 1.84 ± 0.33 *L* s^−1^; *p* = 0.006749), *f* increased significantly (52 ± 9 Hz versus 57 ± 7 Hz; *p* = 0.01869), *t*_upward_ increased slightly (0.2413 ± 0.1537 s versus 0.3276 ± 0.3274 s) but not significantly (*p* = 0.4196) and *U*_upward_ increased significantly (5.06 ± 3.38 *L* s^−1^ versus 6.72 ± 1.45 *L* s^−1^; *p* = 0.01273). The graphing and data analysis software KaleidaGraph version 4.5.2 (Synergy Software) was used to run the above Student's *t*-tests with a significance level of *p* < 0.05.

### 3.4. Encounter and capture of diatom chains by *Centropages* sp.

**Figure 6.**
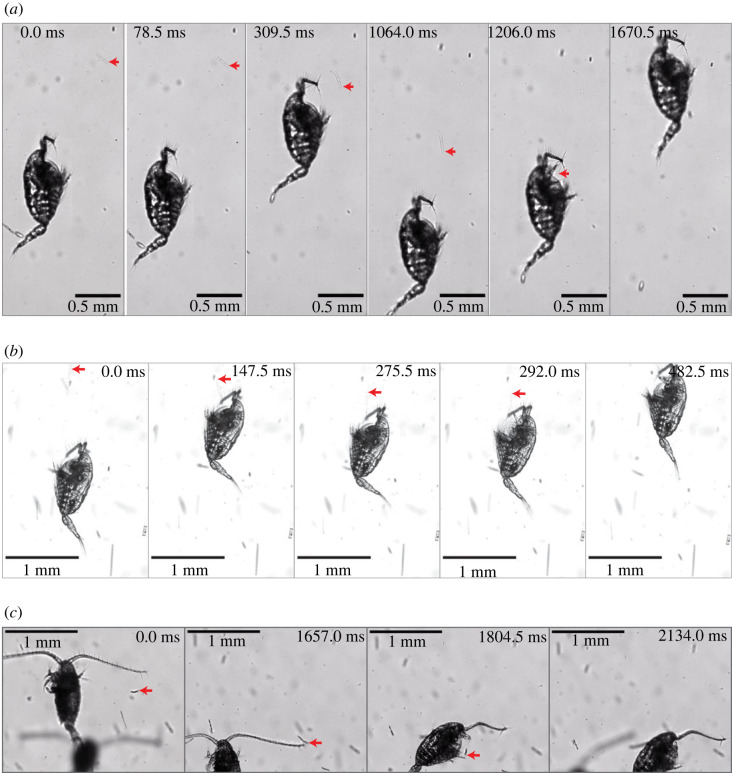
Three time-course image sequences illustrating the detection and manoeuvring for capture of diatom chains by the copepod *Centropages* sp. (*a*) A late copepodite of *Centropages* sp. in swim-and-sink detecting, manoeuvring and capturing a diatom chain (electronic supplementary material, video S2); (*b*) an adult female of *Centropages* sp. swimming upward, pausing to sink briefly and then capturing a diatom chain (electronic supplementary material, video S3) and (*c*) an adult female of *Centropages typicus* in swim-and-sink detecting a diatom chain and then turning to intercept and capture it (electronic supplementary material, video S4). The red arrow in each frame shown before capture points to the diatom chain to be captured.

The present HSMIS observations have revealed connections between the swim-and-sink behaviour and the encounter and capture of diatom chains by *Centropages* sp. Three observed events are described here to demonstrate this. In event 1 (figure 6*a*; electronic supplementary material, video S2), a late copepodite of *Centropages* sp. (*L* = 0.82 mm) first sank (0–78.5 ms) and then swam upward at approximately 3.5 mm s^−1^ (78.5–309.5 ms) and stopped right before bumping into an obliquely oriented diatom chain (at 309.5 ms; reaction distance approx. 0.23 mm); immediately, the copepodite started to sink at approximately 1.7 mm s^−1^; accompanying the sinking motion of the copepodite (309.5–1064.0 ms), the originally obliquely oriented diatom chain was rotated to become vertically oriented; then, the copepodite swam upward at approximately 3.1 mm s^−1^ and this time captured the now vertically oriented diatom chain in no time (around the time-point of 1206.0 ms).

In event 2 (figure 6*b*; electronic supplementary material, video S3), an adult female of *Centropages* sp. (*L* = 0.89 mm) swam upward at approximately 5.3 mm s^−1^ (0–147.5 ms), ending up with touching a cluster of two diatom chains—an obliquely oriented short chain and a nearly vertically oriented long chain; immediately, the female paused to sink only briefly (147.5–275.5 ms) and then swam upward to ingest the vertically oriented long diatom chain. It took approximately 181 ms for the copepod to swallow the long diatom chain.

In event 3 (figure 6*c*; electronic supplementary material, video S4), an adult female of *Centropages typicus* (*L* = 0.85 mm) performed multiple bouts of the swim-and-sink motion (0–1657.0 ms), during which a distal seta located on the left-side antennule (A1) of the copepod touched a diatom chain multiple times; eventually, the copepod turned to precisely intercept and capture the diatom chain.

### 3.5. Flow generated by *Centropages* sp. in swim-and-sink

**Figure 7.**
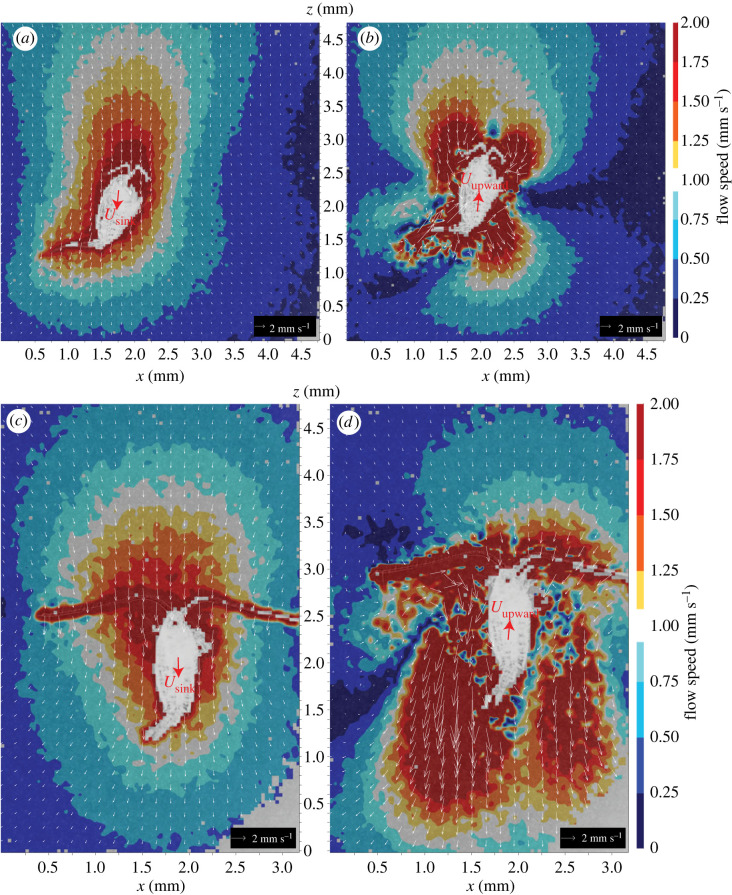
Time-resolved micro-particle image velocimetry (TR-μPIV) measurements of the instantaneous flow fields imposed by a copepodite of *Centropages* sp. in (*a*) free sinking and (*b*) upward swimming (seen from a lateral view; electronic supplementary material, video S5), and by an adult female of *Centropages typicus* in (*c*) free sinking and (*d*) upward swimming (seen from a slightly oblique dorsal view; electronic supplementary material, video S6). A stationary frame of reference is used.

When a copepod of *Centropages* sp. switches between sinking freely and swimming upward, its imposed flow field also alternates almost instantaneously—within 20 ms—between two distinctly different flow patterns, i.e. a sinking current and a swimming current. Here, two events are described: in event 1 (electronic supplementary material, video S5), a copepod, seen from a lateral view, first sank freely at approximately 2.4 mm s^−1^, imposing simultaneously a sinking current that dragged and sheared the fluid from above the copepod (figure 7*a*); since the copepod leaned anteriorly and flexed its urosome toward the dorsal side to sink slightly obliquely, the axis of the sinking current tilted slightly toward the anterior-ventral side of the copepod. Then, the copepod swam upward at approximately 7.4 mm s^−1^ and generated a swimming current that was narrow and short ranged in the region anterior to and above the copepod (figure 7*b*). In event 2 (electronic supplementary material, video S6), a copepod seen from a slightly oblique dorsal view, first sank freely at approximately 2.5 mm s^−1^ and then swam upward at approximately 7.7 mm s^−1^, imposing a sinking current that dragged and sheared the fluid from above the copepod (figure 7*c*) and a narrow, short-ranged swimming current anterior to and above the copepod (figure 7*d*).

## 4. Discussion

The swim-and-sink behaviour of a negatively buoyant copepod has long been claimed as an energetically efficient mode of swimming about a fixed depth [23]. The present CFD simulation results, however, show the opposite: a negatively buoyant copepod that switches repeatedly between upward swimming and free sinking about a fixed depth always requires more mechanical power than that the same copepod that otherwise hovers does. The discrepancy is rooted in the fact that the present study has considered a swimming copepod as a self-propelled body while the previous study used the drag equation for a towed copepod in all calculations. For a self-propelled copepod in steady upward swimming/hovering/partial sinking/free sinking, the present study has derived a unified scaling relationship that relates the body drag to both the excess weight of the copepod and the drag coefficient of the copepod when it is towed (equation 3.1), which is completely different from the drag equation for a towed copepod (equation 3.2). Thus, the drag equation for a towed body cannot be reliably used in calculations concerning a negatively buoyant, self-propelled, swimming copepod.

The present CFD-based modelling of the swim-and-sink behaviour of copepods is a general parametric study. Although no observed events of the copepod *Centropages* sp. were specifically modelled, the CFD-based modelling results presented in figure 2 can be extrapolated to *U*_upward_ / *U*_sink_ = 3 to crudely estimate the mechanical power required by the swim-and-sink behaviour of *Centropages* sp., based on the data of size and swim-and-sink kinematics measured for *Centropages* sp. (table 2). It seems that the swim-and-sink behaviour of *Centropages* sp. probably requires two–four times more mechanical power than that required by hovering. Thus, the swim-and-sink behaviour is energetically costly and thereby likely to be adaptive. On the other hand, compared with hovering, the fast and quickly alternating movements of swim-and-sink make the copepod more apparent to a visually feeding predator such as a larval fish [36,37]. Hence, the advantage such as achieving a more effective feeding should be greater than the disadvantage of being more visually noticeable. Among the various motion behaviours exhibited by copepods, it seems that the mechanical power requirement increases in the following order: sink freely < sink partially < hover < swim-and-sink < escape. The energetically most costly escape jump brings the copepod the highest adaptive advantage, i.e. to avoid being killed by a predator. Thus, it can be argued that an energetically more costly behaviour should have a higher adaptive advantage. (Note that larval fish have also been demonstrated to use ram-and-suction as a hydrodynamically stealthy feeding strategy [38]; thus, an alternative hypothesis may be that the copepods use swim-and-sink to counteract such stealthy predators by preventing the predator from aiming at the prey.)

The results of the present HSMIS behaviour observations and TR-μPIV flow measurements may be interpreted as that *Centropages* sp. uses the swim-and-sink behaviour to perceive and manoeuvre for capture of diatom chains. In an upward swimming phase, the copepod swims at a speed two–three times greater than its terminal sinking speed, generating a narrow, short-ranged swimming current in the region anterior to and above the copepod. This is consistent with a previously proposed distinction between the feeding current and the swimming current [8,25]: when it swims at a speed less than a small fraction (e.g. 1/4) of its terminal sinking speed, a negatively buoyant copepod generates a wide, cone-shaped feeding current. By contrast, when it swims at a speed greater than its terminal sinking speed, a negatively buoyant copepod generates a narrow, short-ranged swimming current. Thus, when viewed in a frame of reference fixed on the copepod body, the narrow, short-ranged swimming current enables the copepod to quickly scan the fluid anterior to and above itself within one–two body lengths (fig. 7*b* of [25]), considering that the upward swimming time was 0.2413 ± 0.1537 s (*n* = 26) at 20°C (table 2*b*). This should allow the copepod to perceive the presence, shape and orientation of an embedded diatom chain, presumably via chemoreception and perhaps in combination with mechanoreception (figure 6*a*; electronic supplementary material, video S2). It can be imagined that, toward the copepod, a horizontally oriented diatom chain would leave behind a curtain-shaped active space, an obliquely oriented diatom chain would leave behind a tilted curtain-shaped active space, and a vertically oriented diatom chain would leave behind a slender active space, thereby betraying its orientation to the copepod. The chemoreception is based on the concept that an alga leaks chemicals that diffuse to set up an active space and that such an active space is deformed in a predictable way in the low Reynolds number laminar flow field generated by a swimming and feeding copepod [10,39]; this concept has been tested experimentally [40] and computationally [41,42]; there was, however, a debate on the feasibility of such chemoreception [43,44]. The mechanoreception is based on the idea that the motion of a copepod may perturb an embedded alga to generate a hydrodynamic signal detectable to the copepod, e.g. a swimming copepod pushing an inert particle away and subsequently attacking and capturing it [45], and a copepod beating its cephalic appendages to generate an oscillating near-field feeding current that causes a phase shift between the fluid velocity and the velocity of an embedded particle, thereby imposing a hydrodynamic signal detectable to the copepod [46]; a theoretical synthesis of hydromechanical signals in the plankton has been conducted, primarily relying on towed body models [47]. These previous studies have put the present observations in a general framework of chemoreception and mechanoreception; however, further, more specific investigations are still needed to elucidate the underlying mechanisms.

Once encountering a diatom chain, the copepod pauses to enter a free sinking phase, during which the sinking current imposed by the copepod rotates the diatom chain appropriately into vertically oriented. The copepod manoeuvring the orientation of the diatom chain is precisely achieved by the unsteady, sheared sinking current that has a slightly tilted axis toward the anterior-ventral side of the copepod. Then, the copepod swims upward to capture the now vertically oriented diatom chain. Comparing the two observed events in which a swim-and-sink copepod captured a diatom chain (figure 6*a* (electronic supplementary material, video S2) versus figure 6*b* (electronic supplementary material, video S3)), the sinking phase in the latter event lasted a much shorter time than in the former event. One possible interpretation of this difference could be that the diatom chain was already approaching a nearly vertical orientation at the beginning of the latter event. It is important to note that this is not a definitive conclusion but rather a potential interpretation based on two observations. Additionally, although sinking is passive, when to stop sinking is actively controlled by the copepod; thus, the copepod adjusting the orientation of the diatom chain can be considered as an active ‘manoeuvring’ process.

A few previous studies have provided evidence that handling and capture of diatom chains are more behaviourally involved than handling and capture of near-spherical algae. When feeding on a chain of *Lauderia borealis*, a tethered female copepod *Eucalanus crassus* spent time to reorient the chain to be in a 90° angle toward its mouth; when encountering a chain of three cells of *Rhizosolenia indica*, a free-swimming female *E. pileatus* repositioned itself such that the chain was also in a 90° angle toward its mouth [48]. The freshwater copepod *Diaptomus sicilis* performed a complicated handling behaviour for feeding on the colonial chain-forming diatom *Melosira italica* [49]. When elongated diatoms (approx. 1 mm long) were entrained into the feeding current generated by the copepod *Temora longicornis*, the diatoms were rotated to align with their long axis parallel to streamlines; however, the fate of the diatoms was not reported [50]. The feeding-current feeding copepod *Temora stylifera* displayed longer feeding bouts, lower appendage beat frequency, and shorter grooming events when offered solitary diatoms than when offered diatom chains [51].

Finally, the swim-and-sink behaviour of copepods is regarded as an innate behaviour in the sense of Tinbergen [52], and Tinbergen's four questions [53] are used to guide a further discussion on the behaviour:

(i) Function: why does the copepod perform the swim-and-sink behaviour? In other words, how does the behaviour increase the fitness of the copepod? This study shows that, compared with hovering, the swim-and-sink behaviour is not an energetically efficient swimming behaviour but rather costly. Furthermore, based on detailed behaviour observations, this study proposes a hypothesis that the swim-and-sink behaviour aids the copepod to perceive and manoeuvre for capture of diatom chains, i.e. allowing better food gathering.

An existing idea about the sinking phase of the swim-and-sink behaviour is that the copepod stops swimming to reduce its self-generated noise so it can ‘listen’ to its surroundings and gather additional information about the object of interest, thereby being able to respond more accurately. As far as perceiving an immobile alga is concerned, however, this idea of ‘listening’ is probably unlikely to work because of the low Reynolds number flow environment. When the active movement of the copepod stops, almost every related process—transporting the fluid for scanning, elongating the active space for chemoreception and perturbing an embedded particle to generate a hydrodynamic signal for mechanoreception—will halt almost immediately, except for diffusion. The diffusion length scale, defined as $\sqrt{4\kappa t_{\mathrm{sink}}}$ where κ (approx. 1.0 × 10^−9^ m^2^ s^−1^) is the diffusion coefficient of chemicals leaked from the alga, is approximately 46 µm, which is much shorter than *U*_sink_ × *t*_sink_ (based on the data presented in table 2). Thus, diffusion is unable to keep pace with the sinking copepod, so a diffusion-based chemoreception of entrained algae will not work either. What remains are the fluid dynamic effects of the copepod's sinking flow on the entrained algae, as well as the copepod's ability to adjust its own orientation while sinking. In fact, diatom chains show preferential horizontal orientation in the ocean [24]; therefore, a handling behaviour is required for copepods—that are usually vertically oriented—to manoeuvre the diatom chains for capture and ingestion. Another scenario could be that those copepods perform the swim-and-sink behaviour instinctively to turn horizontally oriented diatom chains into vertically oriented in a collective sense, thereby benefiting the feeding of those copepods collectively.

**Figure 8.**
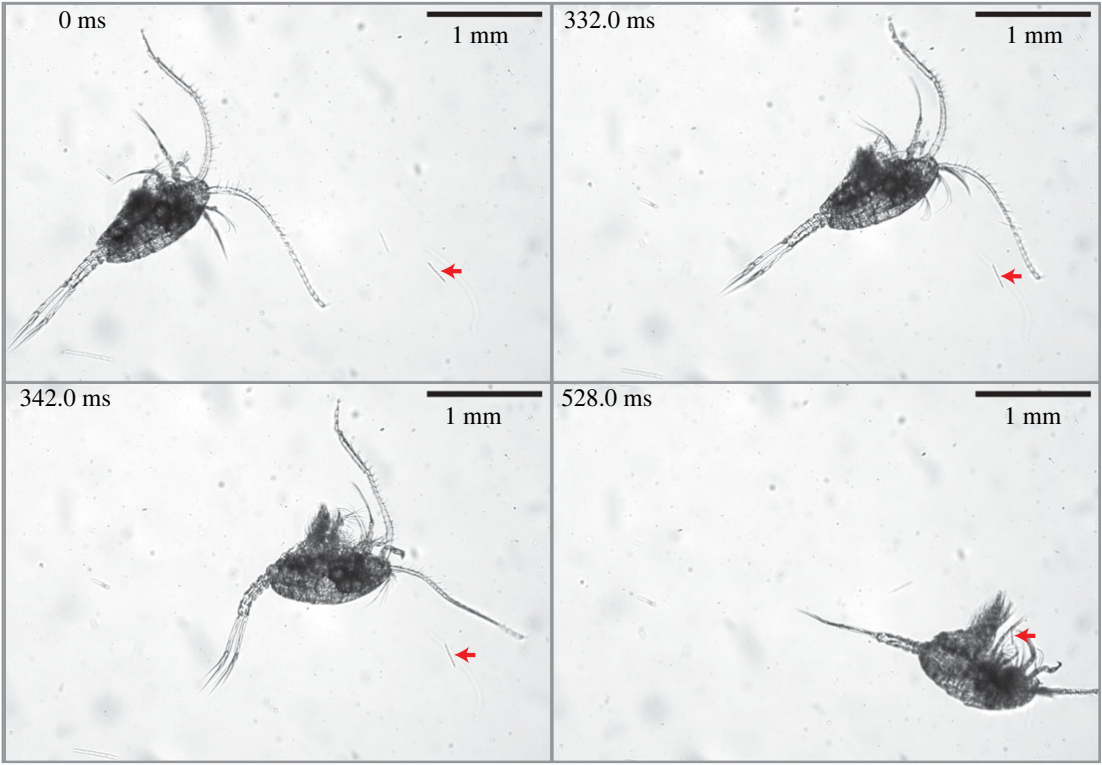
Time-course image sequence illustrating a copepod of *Temora* sp. perceiving and repositioning by a small jump to capture a diatom chain (electronic supplementary material, video S7). From 0 to 332.0 ms, the copepod swam obliquely at approximately 4.8 mm s^−1^, during which the distal part of one antennule (A1) of the copepod swept past the diatom chain. There was no physical contact between the A1 and the diatom chain; however, the copepod seemed to perceive the diatom chain remotely, presumably via chemoreception. Subsequently, the copepod repositioned itself by a small jump and captured the diatom chain with high precision movement of its mouthparts. The red arrow in each frame points to the diatom chain to be captured.

Previous experiments have shown that food conditions affect the feeding movements of copepods. For example, the copepod *Centropages typicus* allocated its time differently between periods of active mouthpart movement and periods of no mouthpart movement, depending on the species and concentration of food available to the copepod [21]. On the other hand, a negatively buoyant copepod may hover to generate a wide, cone-shaped feeding current [10,12] that maximizes its scanned volume of water per unit mechanical power expenditure [25]. Therefore, hovering perhaps occurs more often at lower food concentrations when it is necessary to scan a large volume of water. In contrast with hovering, an alternative hypothesis can be proposed suggesting that the swim-and-sink behaviour confers adaptive advantages in the presence of higher food concentrations; however, the individual-level mechanisms are not so obvious.

(ii) Evolution: how has the swim-and-sink behaviour changed over evolutionary time? This is a difficult question that may be tackled only by investigating different yet closely related species. Not all copepods use the swim-and-sink behaviour to handle and capture diatom chains. For example, the copepod species *Temora* spp. are known to create feeding currents for acquiring algal cells [12]; however, an unpublished personal observation using the same method as the present study has shown a stunning, previously unknown behaviour of *Temora* sp. for capturing a diatom chain: the copepod performed a highly precise repositioning manoeuvre to successfully capture a remotely detected diatom chain (figure 8; electronic supplementary material, video S7). This behaviour shares similarity with a behaviour observed for the non-feeding-current feeding *Centropages* sp. (figure 6*c*; electronic supplementary material, video S4). (Here, *Centropages* sp. is considered a non-feeding-current feeding copepod as it does not generate a wide, cone-shaped feeding current (figure 7) because of its upward swimming speed being two–three times greater than its terminal sinking speed [8,25]; it uses the swim-and-sink behaviour to encounter and capture algae (figure 6).) That is, both copepod species can perform a well-aimed repositioning manoeuvre to precisely adjust the orientation of a perceived diatom chain for a successful capture.

(iii) Causation: what are the proximate causes of the swim-and-sink behaviour? This question remains largely unanswered; however, the copepod may use chemoreception and perhaps in combination with mechanoreception to perceive the presence, shape and orientation of a diatom chain. The copepod needs such information to perform the swim-and-sink behaviour precisely, thereby being able to capture a diatom chain successfully.

(iv) Development: how does the swim-and-sink behaviour develop over the lifetime of an individual copepod? It seems that only copepodites and adult copepods perform the swim-and-sink behaviour. Because of their small size and nearly neutral buoyancy, copepod nauplii sink too slowly to perform the swim-and-sink behaviour in a real sense. In fact, nauplii of subtropical calanoid copepods move nearly continuously, while nauplii of cyclopoid copepods move occasionally [54]. Generally, the swimming behaviour of calanoid nauplii ranges from swimming intermittently to continuously, with late nauplii having behaviours similar to copepodites within some species [55,56]. When feeding on an elongated algal cell of *Rhizosolenia alata*, a tethered nauplius of *Eucalanus crassus* spent more than 1 s to repeatedly adjust the cell to a favourable ingestion angle [57]. By contrast, it took only a small fraction of 1 s for a free-swimming nauplius of *E. pileatus*—that adopted a horizontal orientation and generated a vortical feeding current—to quickly reposition an elongated cell of *R. alata* for ingestion [32]. For nauplii of other copepod species, however, how they handle and capture diatom chains and elongated algae remains an interesting question to explore in the future.

## Data Availability

Original data of the swimming kinematics of copepods are included as electronic supplementary material, section S3 [58].
